# Using a Sensor-Embedded Baseball to Identify Finger Characteristics Related to Spin Rate and Pitching Velocity in Pitchers

**DOI:** 10.3390/s24113523

**Published:** 2024-05-30

**Authors:** Ming-Chia Yeh, Wen-Wen Yang, Yu-Hsuan Hung, Ya-Chen Liu, Jung-Tang Kung, Hsi-Pin Ma, Chiang Liu

**Affiliations:** 1 Department of Exercise Science and Athletic Training, Slippery Rock University, Slippery Rock, PA 16066, USA; mingchia.yeh@sru.edu; 2 Graduate Institute of Sports Equipment Technology, University of Taipei, Taipei 111036, Taiwan; zero1374875@gmail.com; 3 Department of Sports Medicine, China Medical University, Taichung 404327, Taiwan; norman198571@gmail.com; 4 The Office of Physical Education, Chung-Hua University, Hsinchu 300015, Taiwan; yazhen@chu.edu.tw; 5 Department of Sports Training Science—Balls, National Taiwan Sport University, Taoyuan 333325, Taiwan; kung@ntsu.edu.tw; 6 Department of Electrical Engineering, National Tsing Hua University, Hsinchu 300044, Taiwan; hp@ee.nthu.edu.tw

**Keywords:** pitching finger force, rate of force development, pinch strength

## Abstract

Background: Previous investigations have shown a positive relationship between baseball pitching velocity and the kinetic chain involved in pitching motion. However, no study has examined the influence of finger characteristics on pitching velocity and rate of spin via a sensor-embedded baseball. Methods: Twenty-one pitchers volunteered and were recruited for this study. An experimental baseball embedded with a force sensor and an inertial measurement unit was designed for pitching performance measurement. Finger length and strength were measured as dependent variables. Spin rate and velocity were independent variables. Pearson product–moment correlations (r) and intraclass correlation coefficients (ICCs) determined the relationship between finger characteristics and pitching performance. Results: Finger length discrepancy, two-point pinch strength, index finger RFD (rate of force development), middle finger impulse, and force discrepancy had significant correlations with spin rate (r = 0.500~0.576, *p* ≤ 0.05). Finger length discrepancy, two-point pinch, three-point pinch strength, index and middle finger RFD, middle finger impulse, and force combination had significant correlations with fastball pitching velocity (r = 0.491~0.584, *p* ≤ 0.05). Conclusions: Finger length discrepancy, finger pinch strength, and pitching finger force including maximal force and RFD may be factors that impact fastball spin rate and fastball pitching velocity.

## 1. Introduction

Pitching is a series of continuous body movements and has been developed with different pitch types such as fastballs (four-seam and two-seam, cutter, and forkball), breaking balls (curveball, slider, screwball), and changeups (changeup, straight change, circle changeup) based on how the fingers hold the ball [1,2]. These different types of pitches create various movement patterns and are used to throw off the timing of the hitter. During the pitching motion, the lower limbs generate energy and transfer it to the upper extremities through the trunk, which requires sophisticated neuron–muscular coordination to effectively produce force and convey spin and fastball velocity to propel the ball [3]. The whole pitching motion includes six phases: windup, early cocking, late cocking, acceleration, deceleration, and follow-through, with the ball released at the end of the acceleration phase [4].

Spin rate [5] and fastball velocity [6] are two pitching metrics to evaluate a pitcher’s performance level. Studies have shown that the mechanics of pitching may affect fastball pitching performance including fastball velocity [7,8], while findings related to spin rate remain scarce, which may partly be due to pitching capture technologies not being available until most recently, with Ropsodo first released in 2016. The relationship between fastball pitching performance and big muscle groups (e.g., quadriceps, hamstrings, hip rotators, core musculature, and shoulders) involved in the kinetic chain was evaluated via dynamometers and force plates and showed positive correlations because the energy is transferred from the lower limbs to the torso to the upper extremities [9,10]. In addition, angular velocity in various joint movements (e.g., knee flexion, elbow extension, shoulder external rotation) has been shown to be associated with fastball velocity [7,11]. Furthermore, anthropometric characteristics such as body height and humeral and radial length are associated with fastball pitching performance [11]. Regardless of pitching type, towards the end of the pitching kinetic chain, the fingers and thumb apply the last forces to the ball. Although the muscles of the fingers and thumb are relatively smaller compared to the major muscle groups used to pitch, finger and thumb forces are the key factors in creating various ball movement patterns and may play an important role in the determination of spin rate and fastball pitching velocity.

Fingers are capable of dexterous movements [12]. Along with the palm and wrist, the hand’s complex consists of 27 bones, 27 joints, and 28 muscles, as well as numerous ligaments, tendons, blood vessels, nerves, and soft tissue, from an anatomical aspect [13,14]. The movements of fingers and thumbs include flexion, extension, adduction, abduction, and opposition [15]. During a throwing motion, fingers and thumb are the last appendages contacting the ball before releasing it to the target. Previous studies investigated finger characteristics related to pitching performance including finger length, finger angular velocity, and finger strength. Due to fingers’ nimble characteristics and capability for multi-directional movements, they may not only accelerate the velocity of the ball but also allow the ball to spin with different movement patterns [16]. However, a recent study failed to find a significant relationship between finger characteristics and ball spin rate in Major League Baseball (MLB) pitchers. Instead, their findings supported a significant relationship between wrist strength and spin rate, with a larger relative variance (r^2^ = 0.24) [17]. On the other hand, another study revealed that ball velocity increases with stronger finger strength (higher resultant and shear forces, r^2^ = 0.41~0.73) in collegiate baseball players [18].

To date, while the relationship between larger muscle groups and fastball velocity and spin rate has been identified, the relationship between finger characteristics and fastball pitching performance has only been investigated in limited research groups [17,19], with inconsistent results. In addition, there is a lack of studies focused on finger strength and fastball pitching performance, especially in spin rate-related research. Thus, the purpose of this study was to investigate the relationship between finger characteristics (finger length, finger pinch strength, and finger force during pitching) and fastball pitching performance (spin rate and fastball pitching velocity). We hypothesized that finger characteristics, such as finger length, finger strength, and pitching finger force, would have positive relationships with pitching performance, specifically fastball spin rate and velocity.

## 2. Materials and Methods

### 2.1. Experimental Approach to the Problem

**Figure 1:**
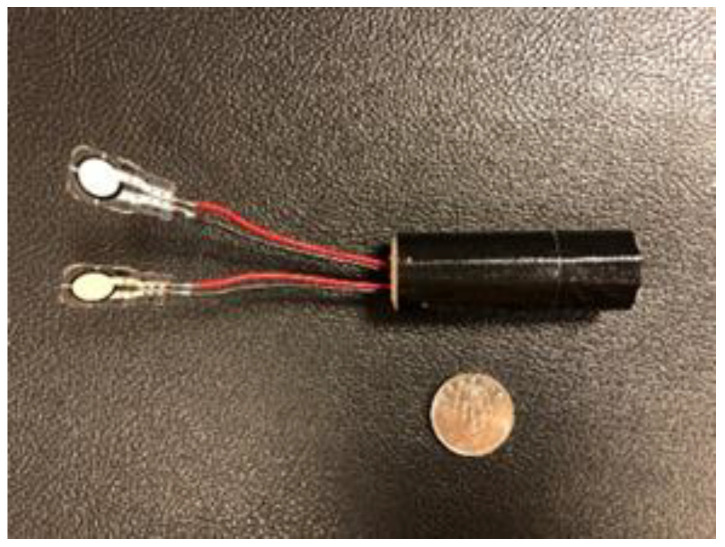
Two force sensors were wired to a customized inertial measurement unit (IMU).

**Figure 2:**
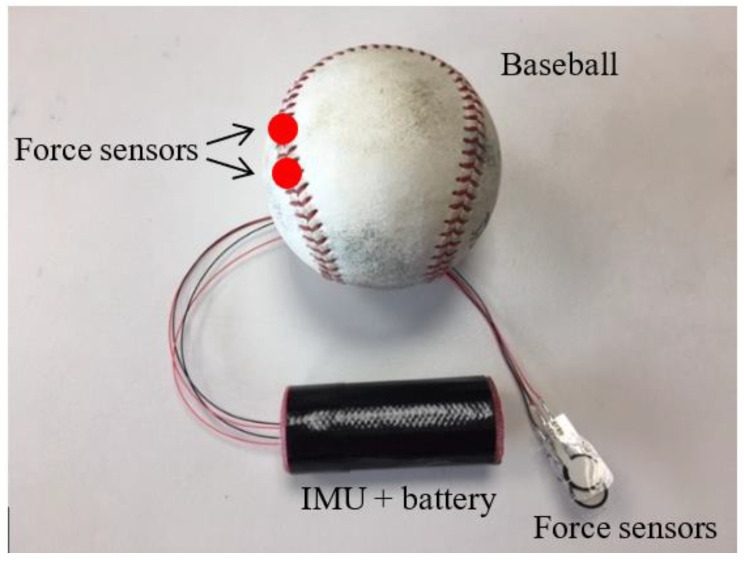
Force sensors and IMU were embedded in an experimental baseball. Red spots refer to where the force sensors were embedded.

The experimental baseball used in this study had a minuscule single-axial force transducer with 433 Hz of recorded frequency (model: Flexi Force A301, Boston, MA, USA) embedded in a standard Taiwan college league baseball (Sakurai 990, Kao-Hsiung, Taiwan) that was designed to obtain the index and middle finger forces during a throwing ball motion (Figure 1 and Figure 2). The FlexiForce A301 is a piezoresistive force sensor (length: 25.4, width: 14, thickness: 0.2, sensing area in diameter: 9.5, unit: mm) for force sensing ranging 0~110 N. This experimental baseball was cut in half and the compressed cork, yarn, and cotton were partially taken out of the baseball. The single-axial force resistor with Bluetooth technology, consisting of a 3-axis accelerometer, 3-axis gyroscope, 3-axis magnetometer, and battery, was placed in the middle of the ball with epoxy coating. Two force pads were attached beneath the leather ball cover and wired to the force resistor. During fastball pitching, the pitchers placed their fingers on the seams of the ball, where we put markers to identify the two force sensors. Additional yarn was re-winded, so the weight of this experimental ball was identical to that of a standard baseball, which is between 142 and 149 g. Thereafter, the two half portions were sealed with styrene–acrylic polymers to ensure the firmness of the experimental ball. For shockproof verification, we threw the experimental baseball to hit the wall directly with a velocity of 100 km/h, and the sensors could still function correctly. Inertial property consistency for the shockproof device revealed that the root mean square errors were all below 1% [20].

### 2.2. Subjects

Twenty-one male, Taiwanese, college baseball pitchers (mean ± SD: age = 20.04 ± 1.04 years; height = 176.71 ± 5.33 cm; weight: 77.61 ± 8.23 kg) were recruited and volunteered for this pitching study. The average number of baseball training years was 9.00 ± 1.84 years. Inclusion criteria included no major injury history of the upper extremities in the past 6 months and the ability to perform best-effort pitching and maximal pinch and grip strength tests. Informed consent was provided prior to the investigation and all subjects understood the potential risks and benefits of this study. This study was conducted according to the tenets of the Declaration of Helsinki and approved by the Institutional Ethics Committee at the University of Taipei (IRB-2020-022).

### 2.3. Procedures

Finger length, finger pinch strength, pitching finger force, hand grip strength, and pitching performance (spin rate and fastball velocity) were measured and performed. The detailed descriptions are below.

### 2.4. Finger Length

The length of the thumb, index, and middle finger of the dominant pitching hand was measured after a photocopy was made of each pitcher’s hand. Each subject was instructed to place their palm on the center of a multifunctional printer (Ricoh MP C2003SP, Tokyo, Japan) with their fingers together. They were not allowed to move their body or hand during scanning. Once the photocopy had been made, the length of each finger was measured twice by research personnel. Each measure was recorded to the nearest 1 mm. If more than 2 mm of difference between two measures was found, another research personnel performed the measurement and averaged the two closest values obtained from the two research personnel to determine the finger length. Measuring finger length via photocopy is popular and reliable due to its convenience, time-saving, and high intraclass correlation coefficients between photocopies and direct measurements [21,22]. One of the cues usually provided by coaches during fastball throwing is to apply more force/pressure to the index and middle fingers. Due to this fact, a variable was created, finger discrepancy, because length difference is one of the finger characteristics and may play a role in applying force to a ball. Thus, finger discrepancy in this study refers to the length difference between the index and middle fingers.

### 2.5. Motor Capacity: Hand Grip Strength

A highly reproducible and valid dynamometer (Takei Kiki Kogyo, Tokyo, Japan) [23] was employed to measure the isometric grip strength of each subject’s dominant pitching hand. Subjects performed the test with their maximal effort in a standing position for three attempts, with a one-minute recovery between each set to prevent the potential risk of fatigue. Verbal encouragement was provided throughout each strength test. The ICC value of the hand grip strength originated from this study was 0.995, indicating excellent reliability.

### 2.6. Motor Capacity: Finger Grip Strength

The participants were instructed to perform a pinching action for each finger strength test, including two-point pinch (thumb and index finger/thumb and middle finger, simultaneously) and three-point pinch (3-jaw grip; thumb, index, and middle fingers). To measure finger strength from each subject’s dominant pitching hand, a PG-60 pinch gauge (B&L Engineering, Tustin, CA, USA) was placed between the pads of the tested fingers [24]. All subjects were set to a standing position with their dominant pitching hand parallel to the ground and performed each finger strength test with their best effort three times, with a one-minute rest in between. Great ICC values between 0.987 and 0.994 were obtained in the finger pinch strength measurements in this study.

### 2.7. Actual Motor Performance: Pitching Finger Force

In sports science, it is important to investigate both motor capacity (e.g., hand grip strength and finger grip strength) and actual motor performance (e.g., pitching finger force). This helps explain the relationship between motor capacity and actual performance to identify if a player needs to emphasize strength development or skill refinement. Rate force development (RFD) evaluates the rate of force development, which has important functional significance in the early phase of fast and forceful muscle contraction. An experimental ball modified from a standard Taiwan college league baseball (Sakurai 990, Kao-Hsiung, Taiwan) embedded with a force transducer was used to obtain the index and middle finger forces during the throwing motion. Each subject was instructed to sit in an upright position with his back leaning against the backrest prior to each throw, with 3 m (9.8 ft) of distance between the cushioning cage and the chair. The tips of the index and middle fingers were placed on the top of the spots where the transducers were located during the throwing motion to capture the finger force data. All subjects threw the experimental baseball to the cage with a four-seam fastball grip and the force was measured while throwing from a seated position. Good to excellent ICC values (0.793~0.953) were found for the finger force test in this study.

### 2.8. Fastball Pitching Performance

To measure spin rate and fastball velocity during each pitch, a popular baseball tracking system (Rapsodo Baseball System, Rapsodo Inc., Fishers, IN, USA), which has been adopted by many MLB teams, was set 0.4 m behind a home plate [25]. The spin rate and fastball velocity were variables taken directly from the Rapsodo output, with the definitions provided in the Rapsodo user manual: velocity indicates how fast a pitch is traveling during ball flight and spin rate refers to the rate at which the ball spins during flight. The home plate was positioned 18.44 m (60 ft 6 in) away from the subject, standing on the pitching rubber, which is identical to the standard pitching distance for baseball games. All participants pitched 5 four-seam fastballs from windup with their best effort to a catcher with 1 min of rest between each pitch in an indoor artificial turf baseball practice setup with a standard Taiwan college league baseball (Sakurai 990, Kao-Hsiung, Taiwan).

### 2.9. Statistical Analyses

Pearson product–moment correlation (*r*) and intraclass correlation coefficients (ICCs) were used to determine the relationship between fastball pitching performance factors (spin rate and fastball velocity) and finger characteristics (finger length, pinch strength, and finger force). A value of *p* ≤ 0.05 was considered statistically significant. Data were analyzed using SPSS (version 22, IBM Statistics, Chicago, IL, USA) and presented as mean values ± standard deviations, unless otherwise specified.

## 3. Results

Descriptive data for all variables are shown in Table 1, including fastball pitching performance and finger characteristics. For finger length characteristics, the results showed that finger length discrepancy had a significant and positive correlation with fastball pitching performance for spin rate (r = 0.58, *p* = 0.02) and fastball velocity (r = 0.53, *p* = 0.03) (Table 2). For finger strength characteristics, a significant and positive correlation between two-point pinch (thumb and middle finger) and fastball spin rate was found (r = 0.51, *p* = 0.03). In addition, three-point pinch also had a significant and positive correlation with fastball pitching velocity (r = 0.49, *p* = 0.04) (Table 2).

For pitching finger force characteristics, index finger RFD showed a significant and positive correlation with fastball spin rate (r = 0.53, *p* = 0.03) and fastball pitching velocity (r = 0.55, *p* = 0.02). In addition, middle finger maximal force during pitching had a significant and positive relationship with fastball spin rate (r = 0.50, *p* = 0.05) and fastball velocity (r = 0.58, *p* = 0.01). Moreover, a significant and positive correlation between middle finger impulse and fastball spin rate (r = 0.56, *p* = 0.03) and fastball velocity (r = 0.55, *p* = 0.03) was found. Furthermore, middle finger RFD (r = 0.53, *p* = 0.04) and force combination (r = 0.51, *p* = 0.04) showed a significant and positive correlation with fastball velocity. Lastly, a significant positive relationship between force discrepancy and fastball spin rate was found (r = 0.52, *p* = 0.04) (Table 2).

## 4. Discussion

The main finding of this study is that pitching-hand finger characteristics are associated with fastball pitching performance. First, finger discrepancy showed a significant positive correlation with both fastball spin rate and fastball velocity, indicating that the difference in finger length may be advantageous for fastball pitching performance. Second, while hand grip strength was not associated with fastball spin rate or fastball velocity, the finger maximal force during pitching and two-point and three-point pinch tests showed significant correlations with fastball spin rate and fastball velocity, meaning that greater finger strength is associated with fastball pitching performance. Third, temporal variables such as RFD and impulse of fingers also had significant correlations with fastball pitching performance (spin rate and velocity), which further demonstrated the relationship between finger strength and force development for pitchers.

The current results showed no significant correlation between finger length and spin rate or fastball velocity (Table 2), which is in line with a recent study [17]. A systematic review revealed that finger length, biacromial width, and hand size are associated with throwing speed in handball players in anthropometric aspects [26]. The size of a handball is much larger than a baseball (58–60 cm vs. 22–24 cm), requiring a larger hand size to better control the ball for throwing, which may explain the significant relationship between hand anthropometrics and throwing performance in handball players but not in baseball pitchers. Moreover, professional pitchers in MLB have an average of 10.0 ± 0.5 cm for the second finger and 11.1 ± 0.6 cm for the third finger [17], which are longer than the measurements found in this current study (see Table 1). Thus, it is not surprising that finger length had minimal impact on fastball spin rate and fastball velocity in the current study. Interestingly, our findings revealed a significant positive correlation between finger discrepancy (the length difference between the index finger and middle finger) and fastball spin rate and pitching velocity (Table 2) in Taiwanese baseball pitchers. We infer that it may be advantageous to manipulate the spin rate of a fastball when a larger difference between finger lengths is present [27]. 

The results from this study demonstrated that the finger force applied to the ball, particularly the maximal force of the middle finger, has a positive correlation with spin rate and fastball velocity. Moreover, force combination and force discrepancy between the index finger and middle finger also showed significant correlations with fastball velocity and spin rate (Table 2). Additionally, finger motor capacity (pinch strength) was associated with fastball spin rate and velocity (Table 2), which was not found in a previous study [17]. The results for finger strength (combined three-point pinch strength) in the current study (10.07 ± 1.94 kg) were greater than those for high school baseball players (7.6 ± 1.4 kg) [28] and lower than those for players in Major League Baseball (12.0 ± 1.9 kg) [17], indicating differences in strength development among different ages and baseball levels. Based on these current findings, it is recommended that baseball pitchers engaged in a strength training program not only focus on major muscle groups but also hand muscles.

This work further analyzed temporal variables including RFD and impulse, which are related to sports performances such as explosive strength [29]. Index finger RFD and middle finger impulse were found to be significantly associated with both fastball spin rate and velocity. Moreover, middle finger RFD also had a positive correlation with fastball pitching velocity (Table 2). A higher RFD means that greater power and force are generated under the same load [30]. Previous research reveals that RFD is influenced by various factors such as maximal force, muscle activation, muscle cross-sectional area, and time to reach a given % force [30], which has been shown to be a determinant to enhance throwing performance in baseball [31] and handball [28] players. Impulse is defined as the force applied during a specific time (impulse = force ∗ Δt), which is particularly important in sports involved in the throwing motion, requiring athletes to perform a greater force for as long as possible, such as in handball and baseball [32]. Thus, impulse and RFD are both considered critical strength elements for evaluating sports performance and physical functionality [33]. Since fingertips are the last part of the pitcher’s body in contact with the ball before the ball is released, it is logically reasonable to assume that finger strength may also play a role in influencing fastball spin rate and velocity throwing performance. This current work is the first to demonstrate that finger RFD and impulse positively relate to fastball throwing velocity and spin rate in college baseball pitchers.

A four-seam, the most common type of fastball used by pitchers, which is thrown with greater velocity and spin rate, allows less reaction time for batters, which could cause more swing and misses, resulting in successful pitching. Moreover, a fastball thrown with a higher spin rate also impacts the forces listed above, which subsequently influences the ball trajectory, thus affecting batter performance [5]. Furthermore, foreign sticky substances have been previously used by pitchers in MLB games to enhance their pitching performance by increasing grip and spin rate on the baseball. Because foreign substances are known to increase grip and spin rate, this is considered cheating and, in June 2021, it caused MLB to ban pitchers from using it and required umpires to mandatorily check pitcher’s hands and gloves after recording the third out of an inning. This announcement inevitably reveals that spin rate is a crucial variable for fastball pitching performance. A study conducted by Nagami et al. confirmed that a greater spin rate accompanies faster ball speed in elite pitchers [34]. To date, the relationship between finger characteristics and spin rate has only been investigated by limited research groups. This study reveals that finger length discrepancy, finger pinch strength, and pitching finger force, including maximal force and RFD, are critical factors in fastball spin rate.

There are a few limitations of this study. First, the subjects were not further categorized into different types of pitchers such as starter or middle reliever, and were closer due to the small sample size. Thus, it remains unclear if different pitcher’s roles would have impacted the results. It is possible that finger characteristics and strength may favor a specific type of pitcher at a professional level. Second, only a four-seam fastball was thrown and evaluated due to its popularity amongst pitchers. However, it is recommended that future studies evaluate different pitch types, such as breaking balls and changeups, as these are additional pitch types thrown during games. Lastly, pitching a baseball is a motion that involves the transmission of a force initially generated from the lower limbs through the core muscles to the upper extremities; however, this study only focused on finger characteristics. Thus, we cannot exclude other potential effects from the recruitment of the whole kinetic chain on fastball pitching performance.

With respect to clinical relevance, unlike previous studies that mostly focused on the torso or larger extremities, this study investigated the relationship between fastball pitching performance and finger characteristics, which are the last and relatively smaller body parts contributing to throwing a baseball. This study revealed that the length difference between the index finger and middle finger has a significant correlation with fastball spin rate. Moreover, not only finger strength but also temporal variables including RFD and impulse were found to be related to fastball spin rate and velocity. Thus, coaches are encouraged to (1) understand what variables may influence spin rate, (2) understand what variables may impact fastball velocity, and (3) integrate finger and hand strength development in the training program to optimize a pitcher’s performance.

## 5. Conclusions

In conclusion, finger length discrepancy, finger pinch strength, and pitching finger force, including maximal force and RFD, may play a role in fastball spin rate and velocity. These findings suggest that besides a well-rounded training program involved in the whole pitching kinetic chain, strength and conditioning coaches may also consider implementing hand/finger resistance training to further develop greater RFD and impulse, which may favor spin rate and fastball velocity for pitchers, optimizing their fastball pitching performance.

## Figures and Tables

**Table 1:** Descriptive statistics for all variables.

Variables	Mean ± SD	Range
Fastball pitching performance		
Spin rate (rpm)	1751.20 ± 171.22	1422.00–2056.00
Fastball velocity (km/h)	125.33 ± 6.49	113.50–138.50
Finger length (cm)		
Thumb	6.13 ± 0.32	5.60–6.90
Index finger	7.24 ± 0.33	6.80–7.80
Middle finger	8.11 ± 0.43	7.40–9.00
Finger discrepancy (index and middle)	0.85 ± 0.17	0.50–1.20
Strength (N)		
Hand grip	506.59 ± 53.79	359.57–573.69
Two-point pinch (index and thumb)	87.70 ± 17.48	57.83–133.45
Two-point pinch (middle and thumb)	78.58 ± 12.85	56.34–97.86
Three-point pinch (index, middle, and thumb)	98.75 ± 19.01	72.65–149.76
Two-point pinch strength discrepancy	14.60 ± 11.20	1.48–41.52
Pitching finger force on baseball		
Index maximal strength (N)	153.20 ± 1.15	149.58–155.23
Index RFD (N/s)	23.82 ± 6.85	12.38–37.49
Index impulse (N × s)	33.63 ± 5.91	19.45–41.00
Time of index applying force on the ball (s)	0.22 ± 0.03	0.12–0.27
Middle finger maximal force (N)	153.10 ± 0.81	151.96–154.56
Middle finger RFD (N/s)	17.94 ± 6.42	8.00–35.74
Middle finger impulse (N × s)	33.14 ± 5.49	24.14–42.50
Time of middle finger applying force on the ball (s)	0.21 ± 0.03	0.16–0.27
Force combination (N)	306.25 ± 1.62	302.18–309.57
Force discrepancy (N)	0.90 ± 0.70	0.08–3.02
Time difference (s)	0.01 ± 0.01	0.01–0.03

Note: rpm = revolution per minute, km/h = kilometers per hour, cm = centimeter, N = Newton, s = second, RFD = rate of force development, finger discrepancy = the absolute value of length difference between index and middle finger, force combination = the summation of force generated by index and middle finger, force discrepancy = the absolute value of force difference between index finger and middle finger, time difference = the absolute value of time difference during the period that each finger (index finger and middle finger) was applying force on the experimental ball while performing pitching.

**Table 2:** Correlation between finger characteristics and fastball pitching performance.

Variables	Spin Rate		Fastball Velocity	
	Pearson’s Correlation	*p* Value	Pearson’s Correlation	*p* Value
Finger length (cm)				
Thumb	−0.23	0.33	0.05	0.84
Index finger	0.08	0.74	−0.11	0.65
Middle finger	0.18	0.47	0.20	0.40
Finger discrepancy	0.58	0.02 *	0.53	0.03 *
Strength (N)				
Hand grip	0.04	0.86	0.27	0.26
Two-point pinch (index and thumb)	0.01	0.98	0.26	0.28
Two-point pinch (middle and thumb)	0.51	0.03 *	0.58	0.01 *
Three-point pinch (index, middle, and thumb)	0.32	0.18	0.49	0.04 *
Two-point pinch discrepancy	−0.23	0.34	0.06	0.82
Pitching finger force on baseball				
Index maximal force (N)	−0.23	0.35	0.24	0.33
Index finger RFD (N/s)	0.53	0.03 *	0.54	0.02 *
Index finger impulse (N ∗ s)	−0.12	0.64	0.06	0.83
Time of index finger applying force on the ball (s)	−0.16	0.55	−0.08	0.77
Middle finger maximal force (N)	0.50	0.05 *	0.57	0.01 *
Middle finger RFD (N/s)	0.23	0.36	0.53	0.04 *
Middle finger impulse (N ∗ s)	0.56	0.03 *	0.55	0.03 *
Time of middle finger applying force on the ball (s)	0.53	0.04 *	0.34	0.18
Force combination (N)	−0.01	0.98	0.51	0.04 *
Force discrepancy (N)	0.52	0.04 *	0.14	0.59
Time difference (s)	0.10	0.69	−0.07	0.79

Note: cm = centimeter, N = Newton, s = second, RFD = rate of force development, force combination = the summation of force generated by index and middle finger, finger discrepancy = the absolute value of length difference between index and middle finger, time difference = the absolute value of the difference between the time of index and middle fingers applying force on the ball. * Significant difference between finger parameter and fastball pitching performance (*p* < 0.05).

## Data Availability

The data presented in this study are available upon request from the corresponding author.

## References

[1] Fleisig G.S., Laughlin W.A., Aune K.T., Cain E.L., Jeffrey R., Andrews J.R., Fleisig G.S. (2016). Differences among Fastball, Curveball, and Change-up Pitching Biomechanics across Various Levels of Baseball. Sports Biomech..

[2] Fleisig G.S., Kingsley D.S., Loftice J.W., Dinnen K.P., Ranganathan R., Dun S., Escamilla R.F., Andrews J.R. (2006). Kinetic Comparison among the Fastball, Curveball, Change-up, and Slider in Collegiate Baseball Pitchers. Am. J. Sports Med..

[3] Fleisig G.S., Barrentine S.W., Zheng N., Escamilla R.F., Andrews J.R. (1999). Kinematic and Kinetic Comparison of Baseball Pitching among Various Levels of Development. J. Biomech..

[4] Seroyer S.T., Nho S.J., Bach B.R., Bush-Joseph C.A., Nicholson G.P., Romeo A.A. (2010). The Kinetic Chain in Overhand Pitching: Its Potential Role for Performance Enhancement and Injury Prevention. Sports Health.

[5] Nagami T., Higuchi T., Kanosue K. (2013). How Baseball Spin Influences the Performance of a Pitcher. J. Phys. Fit. Sports Med..

[6] Fortenbaugh D., Fleisig G.S., Andrews J.R. (2009). Baseball Pitching Biomechanics in Relation to Injury Risk and Performance. Sports Health.

[7] Stodden D.F., Fleisig G.S., McLean S.P., Andrews J.R. (2005). Relationship of Biomechanical Factors to Baseball Pitching Velocity: Within Pitcher Variation. J. Appl. Biomech..

[8] Leenen T., Tright B., Hoozemans M., Veeger D. (2020). Effects of a Disturbed Kinetic Chain in the Fastball Pitch on Elbow Kinetics and Ball Speed. Proceedings.

[9] Mullaney M.J., McHugh M.P., Donofrio T.M., Nicholas S.J. (2005). Upper and Lower Extremity Muscle Fatigue after a Baseball Pitching Performance. Am. J. Sports Med..

[10] Lehman G., Drinkwater E.J., Behm D.G. (2013). Correlation of Throwing Velocity to the Results of Lower-Body Field Tests in Male College Baseball Players. J. Strength Cond. Res..

[11] Matsuo T., Escamilla R.F., Fleisig G.S., Barrentine S.W., Andrews J.R. (2001). Comparison of Kinematic and Temporal Parameters between Different Pitch Velocity Groups. J. Appl. Biomech..

[12] Sikdar S., Rangwala H., Eastlake E.B., Hunt I.A., Nelson A.J., Devanathan J., Shin A., Pancrazio J.J. (2013). Novel Method for Predicting Dexterous Individual Finger Movements by Imaging Muscle Activity Using a Wearable Ultrasonic System. IEEE Trans. Neural Syst. Rehabil. Eng..

[13] Lee K.S., Jung M.C. (2015). Ergonomic Evaluation of Biomechanical Hand Function. Saf. Health Work.

[14] Kaplan D.Ö. (2016). Evaluating the Relation between Dominant and Non-Dominant Hand Perimeters and Handgrip Strength of Basketball, Volleyball, Badminton and Handball Athletes. Int. J. Environ. Sci. Educ..

[15] Panchal-Kildare S., Malone K. (2013). Skeletal Anatomy of the Hand. Hand Clin..

[16] Theobalt C., Albrecht I., Haber J., Magnor M., Seidel H.P. Pitching a Baseball—Tracking High-Speed Motion with Multi-Exposure Images. Proceedings of the ACM SIGGRAPH 2004 Papers, SIGGRAPH 2004.

[17] Wong R., Laudner K., Evans D., Miller L., Blank T., Meister K. (2021). Relationships between Clinically Measured Upper-Extremity Physical Characteristics and Ball Spin Rate in Professional Baseball Pitchers. J. Strength Cond. Res..

[18] Kinoshita H., Obata S., Nasu D., Kadota K., Matsuo T., Fleisig G.S. (2017). Finger Forces in Fastball Baseball Pitching. Hum. Mov. Sci..

[19] Wong R., Laudner K., Amonette W., Vazquez J., Evans D., Meister K. (2023). Relationships between Lower Extremity Power and Fastball Spin Rate and Ball Velocity in Professional Baseball Pitchers. J. Strength Cond. Res..

[20] Hsieh T.-H. (2021). A Smart Baseball with Multi-Sensor Configuration Based on IMU Sensors at a High Sampling Rate. Master’s Thesis.

[21] Manning J.T., Fink B., Neave N., Caswell N. (2005). Photocopies Yield Lower Digit Ratios (2D:4D) than Direct Finger Measurements. Arch. Sex. Behav..

[22] Robinson S.J., Manning J.T. (2000). The Ratio of 2nd to 4th Digit Length and Male Homosexuality. Evol. Hum. Behav..

[23] Balogun J.A., Onigbinde A.T. (1991). Intratester Reliability and Validity of the Takei Kiki Kogo Hand Grip Dynamometer. J. Phys. Ther. Sci..

[24] Vollmer B., HolmströM L., Forsman L., Krumlinde-Sundholm L., Valero-Cuevas F.J., Forssberg H., UlléN F. (2010). Evidence of Validity in a New Method for Measurement of Dexterity in Children and Adolescents. Dev. Med. Child Neurol..

[25] Diffendaffer A.Z., Slowik J.S., Lo N.J., Drogosz M., Fleisig G.S. (2019). The Influence of Mound Height on Baseball Movement and Pitching Biomechanics. J. Sci. Med. Sport.

[26] Vila H., Ferragut C. (2019). Throwing Speed in Team Handball: A Systematic Review. Int. J. Perform. Anal. Sport.

[27] Tajika T., Kobayashi T., Yamamoto A., Shitara H., Ichinose T., Shimoyama D., Okura C., Kanazawa S., Nagai A., Takagishi K. (2015). Relationship between Grip, Pinch Strengths and Anthropometric Variables, Types of Pitch Throwing among Japanese High School Baseball Pitchers. Asian J. Sports Med..

[28] Marques M.C., Saavedra F.J., Marques M.C., Abrantes C., Aidar F.J. (2011). Associations between Rate of Force Development Metrics and Throwing Velocity in Elite Team Handball Players: A Short Research Report. J. Hum. Kinet..

[29] Tillin N.A., Jimenez-Reyes P., Pain M.T.G., Folland J.P. (2010). Neuromuscular Performance of Explosive Power Athletes versus Untrained Individuals. Med. Sci. Sports Exerc..

[30] Maffiuletti N.A., Aagaard P., Blazevich A.J., Folland J., Tillin N., Duchateau J. (2016). Rate of Force Development: Physiological and Methodological Considerations. Eur. J. Appl. Physiol..

[31] McEvoy K.P., Newton R.U. (1998). Baseball Throwing Speed and Base Running Speed: The Effects of Ballistic Resistance Training. J. Strength Cond. Res..

[32] Urbin M.A., Stodden D., Boros R., Shannon D. (2012). Examining Impulse-Variability in Overarm Throwing. Mot. Control.

[33] McGhie D., Østerås S., Ettema G., Paulsen G., Sandbakk Ø. (2020). Strength Determinants of Jump Height in the Jump Throw Movement in Women Handball Players. J. Strength Cond. Res..

[34] Nagami T., Morohoshi J., Higuchi T., Nakata H., Naito S., Kanosue K. (2011). Spin on Fastballs Thrown by Elite Baseball Pitchers. Med. Sci. Sports Exerc..

